# Supplementary material for: A Critical View of the Application of the APEX Software (Aqueous Photochemistry of Environmentally-Occurring Xenobiotics) to Predict Photoreaction Kinetics in Surface Freshwaters
Source: Molecules. 2019 Dec 18;25(1):9. doi: 10.3390/molecules25010009 (PMC7017383; doi:10.3390/molecules25010009)
Supplement: Supplementary file 1 [file molecules-25-00009-s001.zip › 3.2.4_gcc-4.4.0.zip!/3.2.4_gcc-4.4.0/3.2.4_gcc-4.4.0/doc/octave/html/interpreter/Strings.html]

Strings - Untitled


Next: Data Containers,
Previous: Numeric Data Types,
Up: Top


---

## 5 Strings

A string constant consists of a sequence of characters enclosed in
either double-quote or single-quote marks. For example, both of the
following expressions

```
     "parrot"
     'parrot'
```

represent the string whose contents are parrot. Strings in
Octave can be of any length.

Since the single-quote mark is also used for the transpose operator
(see Arithmetic Ops) but double-quote marks have no other purpose in Octave,
it is best to use double-quote marks to denote strings.

Strings can be concatenated using the notation for defining matrices. For
example, the expression

```
     [ "foo" , "bar" , "baz" ]
```

produces the string whose contents are foobarbaz. See Numeric Data Types, for more information about creating matrices.

- Escape Sequences in string constants- Character Arrays- Creating Strings- Comparing Strings- Manipulating Strings- String Conversions- Character Class Functions
